# Clinical Outcomes of the Transforaminal Lumbar Interbody Fusion Technique Among Patients With Low Back Pain Showing Type 1 Modic Changes on MRI

**DOI:** 10.7759/cureus.61745

**Published:** 2024-06-05

**Authors:** Khalid Murrad, Yazeed Al Harbi, Laila M Alsabbagh, Khulood Alwehaibi, Rakan Al Salhi, Waleed Awwad

**Affiliations:** 1 Orthopaedic Surgery, King Saud University, Riyadh, SAU

**Keywords:** spine, ortho surgery, lower back pain, clinical outcomes, transforaminal lumbar interbody fusion

## Abstract

Introduction

The unilateral transforaminal lumbar interbody fusion (TLIF) signifies a different surgical method, circumventing both the anterior method and the method via the spinal canal. Due to the shortage of literature available for clinical outcomes and consequences post-TLIF, we undertook the current study to assess the TLIF technique's clinical outcomes among patients with low back pain showing type 1 Modic changes on MRI.

Material and methods

A cross-sectional study was conducted between January 2019 and March 2021. All patients included in the study had Modic type 1 change and disabling low back pain as the main complaint and/or leg pain. Data were collected on age, body mass index (BMI), gender, and other risk factors like diabetes mellitus, steroid use, and smoking. Pain intensity was evaluated using a visual analog scale (VAS) before and after surgery. A radiographic evaluation was also performed. Pre and post-operative pain scores and differences in disc height were assessed using the Wilcoxon rank sum test. A p-value of less than 0.05 was considered significant.

Results

The mean length of stay in the hospital was 4.3±1.61. The mean pre-operative lower back pain score was 8.78±0.79. The mean post-operative score was substantially lowered to 0.83±0.7. There was a significant difference between pre- and post-operative lumbar pain (p-value < 0.001). There was a significant increase in mean disc height from pre-operative (7.14 mm) to post-operative (11.02 mm) and also at one year (10.21 mm) with a p-value of <0.001. Of the patients, 82.14% did not have any complications, and 3.57% each had either delayed wound healing without any infection or transient post-operative radiculopathy that improved in six weeks.

Conclusion

TLIF procedure can be considered safe to provide anterior and posterior column support by adopting a unilateral posterior approach. The outcomes were favorable in terms of no prolonged length of stay, less blood loss, no mortality, reduction in the severity of pain, and improvement in disc height. However, the appropriate selection of patients for this technique is pivotal for the success of the procedure.

## Introduction

The vertebral endplate is a thin layer that covers the upper and lower parts of the vertebral body separating it from the intervertebral disc. It is made of trabecular bone layers with porous structures along with numerous neural elements [1,2]. The signal intensity of the vertebral body closer to the endplate on magnetic resonance imaging (MRI) changes throughout the process of intervertebral disc degeneration; these changes are known as Modic changes (MCs). Modic et al., in 1988, first defined MCs with three main types [3]. On the findings of MRI, type I MCs can be featured by the reduced signal intensity of the T1-weighted images and augmented intensity of T2-weighted images. In contrast to MC type 1, MC type II is characterized by an augmented signal intensity on T1-weighted images and either isointense or slightly hyperintense intensity on T2-weighted images [3]. MC Type III demonstrates diminished intensity on both T1 and T2 weighted images. Type I MCs can be characterized by the pathological change of edema, Type II MCs by the pathological change of fatty change, and Type III MCs by the pathological changes of fibrosis/sclerosis. These MCs are used as a reliable classification despite various clinical settings and clinicians' experiences [4]. 

Chronic low back pain (LBP) is one of the leading causes of disability worldwide [5]. As MCs occur along with the process of degeneration of the intervertebral disc, patients usually present with pain in their backs as the main complaint. Multiple studies studied the incidence rate of MCs in patients who complained of back pain and underwent an MRI study. The proportion of MC in patients complaining of back pain varies between 8% and 80.1% [6-11]. Almost all studies reported that MC type II is the most observed in their patients [8,11-13]. However, among different types of Modic changes, inflammatory MC type I in the lumbar region is significantly associated with chronic LBP [14]. The involvement of inflammatory mediators including tumor necrosis factor (TNF)-immunoreactive cells has been suggested as the underlying mechanism of LBP [15]. Numerous studies have established that several factors may contribute to the frequency of MCs, including older age, male gender, obesity, long-term hard physical work, and diabetes mellitus. Among these factors, the presence of degenerative disk disease is the most frequently reported risk factor to be associated with MC type I.

The unilateral TLIF signifies a different surgical method, circumventing both the anterior method and the method via the spinal canal. Harms and Jeszenszky first described the TLIF procedure in 1998 [16]. The technique has received great interest over the last few decades with proven evidence regarding its efficacy and versatility [17]. The TLIF technique was first performed in 1997 using a specially designed titanium cage. As with prior posterior and anterior lumbar fusion techniques, TLIF was initially indicated for isthmic and degenerative spondylolistheses, discogenic pain syndromes, and post-discectomy syndromes unresponsive to non-surgical treatments [18]. To contribute to the growing body of literature on TLIF outcomes, this study aimed to assess the clinical outcomes of the TLIF technique among patients with LBP showing type 1 MCs on MRI.

## Materials and methods

This study was conducted at King Saud University, Riyadh, Saudi Arabia, from January 2019 to March 2021. The study was approved by the Institutional Review Board of King Saud University (approval number: KSU-IRB 0023 E). We evaluated data of 35 patients with disabling LBP and/or leg pain who showed MC type 1 on MRI and were candidates for surgery. We excluded patients with a history of trauma to the spine, previous lumbar spine surgery, evidence of lumbar tumors on MRI, congenital anomalies, lumbar vertebral fracture, current radiotherapy, and spinal deformities such as scoliosis that could affect the decision on the surgery type.Basic demographic data were compiled for all patients in the study including age, body mass index (BMI), gender, and other risk factors like diabetes mellitus, steroid use, and smoking.

Clinical and radiological outcome measurements

Pain intensity was evaluated using a visual analog scale (VAS), where 0=no pain and 10=worst pain, prior to and weeks after surgery. A radiographic evaluation was also performed. Anteroposterior and lateral views of radiographic examination were performed pre-operatively, post-operatively once patients were able to stand and mobilize, at the six-week visit, then every three months for the first year. We measured lumbar lordosis and disc space height. We also measured other findings related to surgical outcomes that could affect them, including Pfirrmann grading, estimated blood loss, surgery time, and pre-operative lab values (white blood cell count, total protein, albumin). Additionally, we recorded the length of hospital stay and any postoperative complications.

Statistical analysis

Frequencies and proportions were calculated for categorical variables including gender, comorbidities, complications, blood type, and smoking status. For continuous variables such as age, BMI, disc height, and estimated blood loss, mean and standard deviations (SD) were calculated. A bar chart was generated for the proportion of complications. Also, we compared pre- and post-operative pain scores and differences in disc height using the Wilcoxon rank sum test. A p-value of less than 0.05 was considered significant. All data were analyzed using IBM SPSS Statistics for Windows, Version 24.0 (Released 2016; IBM Corp., Armonk, New York, United States).

## Results

Table 1 displays the sociodemographic characteristics of patients undergoing the TLIF procedure as described by Harms et al. [16]. The mean age of the participants was 48.36 years, with an SD of 10.79 years. The mean BMI was 29.62 with an SD of 3.73. There was an equal proportion of males and females with a 1:1 ratio. Over one-third of the patients (39%) had blood group O positive and one quarter (25%) were A positive. About two-thirds (61%) of the patients’ L4-L5 was involved and one-third (32%) of the patients’ L5-S1 was involved. About 14% of the patients suffered from diabetes mellitus and 11% had dyslipidemia. About one-third of the patients (29%) had a history of hypertension and 11% had hypothyroidism. Less than a quarter of patients (21%) were smokers, and 7% had ischemic heart disease. 

**Table 1:** Sociodemographic characteristics of the patients undergoing transforaminal lumbar interbody fusion (N=28)

Continuous Variables		Mean±SD
Age (years)		48.36±10.79)
BMI		29.62±3.73)
Categorical Variables	Frequency (Percentage)	
Gender		
Female		14 (50%)
Male		14 (50%)
Blood group		
A+		7 (25%)
AB+		1 (4%)
B+		1 (4%)
O+		11 (39%)
Involved segment		
L2-L3		1 (4%)
L3-L4		1 (4%)
L4-L5		17 (61%)
L5-S1		9 (32%)
Pfirrmann grading		
2		1 (3.6%)
3		3 (10.7%)
4		14 (50.0%)
5		10 (35.7%)
Diabetes mellitus		
No		24 (86%)
Yes		4 (14%)
Dyslipidemia		
No		25 (89%)
Yes		3 (11%)
Hypertension		
No		20 (71%)
Yes		8 (29%)
Hypothyroidism		
No		25 (89%)
Yes		3 (11%)
Smoking		
No		22 (79%)
Yes		6 (21%)
Ischemic heart disease		
No		26 (93%)
Yes		2 (7%)
Other comorbidities		
No		22 (78.6%)
Asthma		1 (3.6%)
Benign prostatic hyperplasia		3 (10.7)
Chronic kidney disease		1 (3.6)
Graves’ disease		1 (3.6%)

Table 2 shows the findings regarding the clinical outcomes of the TLIF procedure. The data revealed that the average length of stay in the hospital was 4.3 days with an SD of 1.61. One-quarter of the patients (25%) stayed for seven days, and 39.29% stayed for 11 days in the hospital. The mean pre-operative lower back pain score was 8.78 with an SD of 0.79. About a quarter of patients had severe pain with a score of 8 and 39.3% had severe pain score of 9. However, 14.3% had the worst pre-operative pain with a maximum score of 10. The mean post-operative score was substantially lowered to 0.83 with an SD of 0.7. The majority of the patients (39.3%) had a lower post-operative pain score of 1 and 14.3% had a score of 2. There was a significant difference between pre- and post-operative lumbar pain (P-value < 0.001).

**Table 2:** Clinical outcomes (N=28)

Outcomes	Values	
Amount of drainage, n (%)		
No drain		28 (100%)
Days of removal suction drainage, n (%)		
None		28 (100%)
Intraoperative dural tear, n (%)		
No		28 (100%)
Length of stay (days), mean±SD		4.3±1.61
2 days, n (%)		1 (3.57%)
3 days, n (%)		6 (21.43%)
4 days, n (%)		11 (39.29%)
5 days, n (%)		7 (25.00%)
6 days, n (%)		1 (3.57%)
11 days, n (%)		1 (3.57%)
Preoperative score of lower back pain, mean±SD		8.78±0.97
7, n (%)		1 (3.6%)
8, n (%)		7 (25.0%)
9, n (%)		11 (39.3%)
10, n (%)		4 (14.3%)
One-year follow-up score of lower back pain, mean±SD		0.83±0.7
0, n (%)		8 (28.6%)
1, n (%)		11 (39.3%)
2, n (%)		4 (14.3%)
Surgical time, mean±SD		162.04±52.43
WBC, mean±SD		5.6±1.33
Estimated blood loss (cc), mean±SD		216.4±130
Preoperative lumbar lordosis angle, mean±SD		34.7±10.7
Postoperative lumbar lordosis angle, mean±SD		42.3±9.51
Disc height MRI (mm), mean±SD		7.6±2.2
Preoperative disc height X-ray (mm), mean±SD		7.14±3.86
Postoperative disc height X-ray post-op (mm), mean±SD		11.02±1.55
One-year follow-up disc height X-ray (mm), mean±SD		10.21±5.35

**Figure 1:**
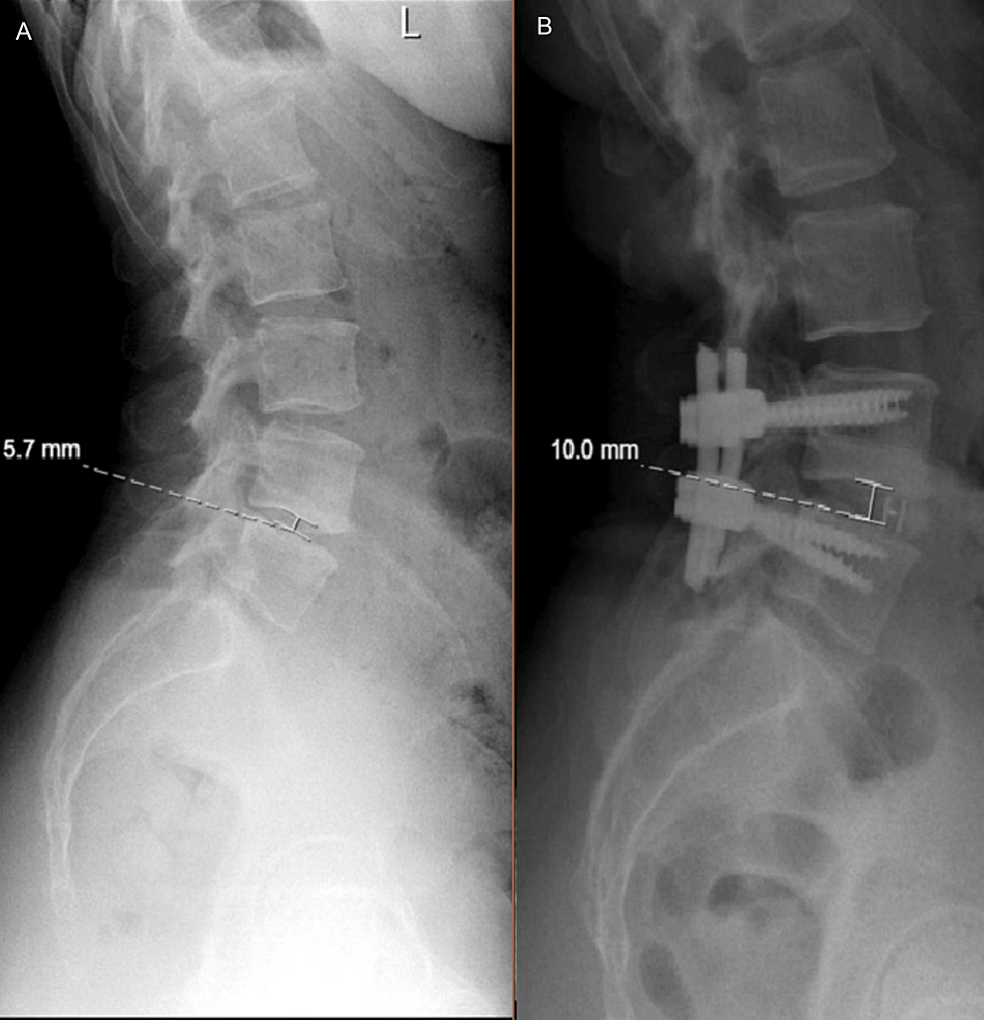
Case example of disc height restoration: (A) preoperative and (B) postoperative

The mean surgical time was 162.04 minutes with an SD of 52.43. The estimated average blood loss was 216.4 cc with an SD of 130 cc. However, no patients had excessive drainage or intraoperative dural tears. The mean lumbar lordosis angle was 34.7° (SD: 10.7°) preoperatively, which significantly increased to a mean of 42.3° (SD: 9.51°) postoperatively (p<0.001). Similarly, the preoperative mean disc height on MRI was 7.6 mm (SD: 2.2 mm), and on X-ray was 7.14 mm (SD: 3.86 mm). There was a significant increase in mean disc height from preoperative (7.14 mm) to postoperative (11.02 mm), and at the one-year follow-up, it was 10.21 mm (p<0.001). An example is shown in Figure 1.

**Figure 2:**
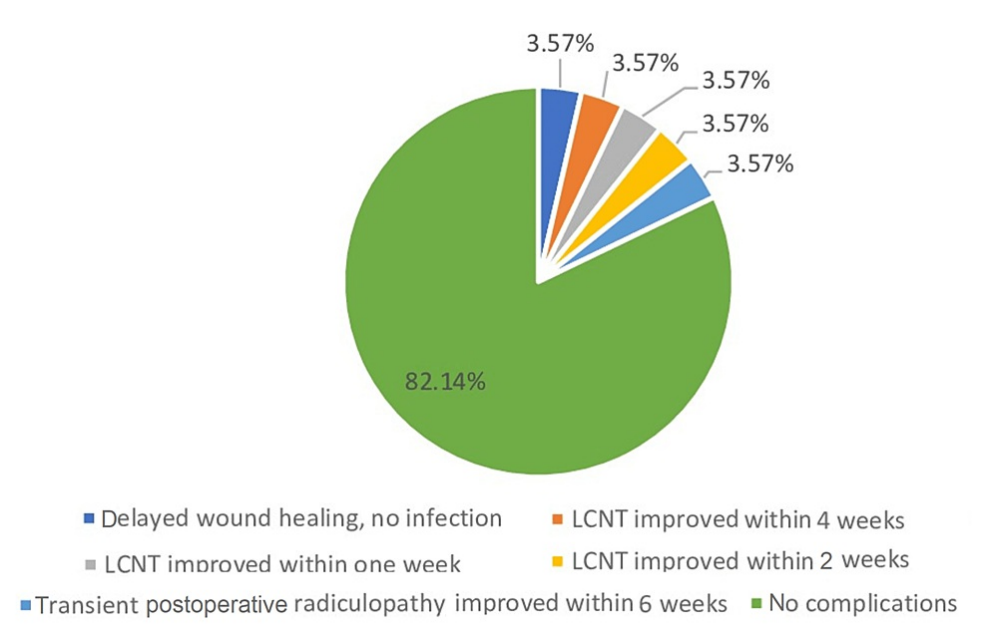
Distribution and type of complications after transforaminal lumbar interbody fusion procedure LCNT: lateral femoral cutaneous nerve of the thigh

Figure 2 shows the proportion of complications after surgery. Of the patients, 82.14% did not have any complications while 3.57% each had either delayed wound healing without any infection, improvement of lateral femoral cutaneous nerve of the thigh (LCNT) within four weeks, improvement of LCNT within two weeks, improvement of LCNT within one week, and transient postoperative radiculopathy that improved in six weeks.

## Discussion

This cross-sectional study assessed the clinical outcomes of the TLIF procedure for patients with LBP showing type 1 MCs on MRI. The results demonstrated successful outcomes, with over 80% of patients experiencing no postoperative complications. Additionally, the average length of hospital stay was approximately four days. Although the mean estimated blood loss was 216 cc, no patients had excessive drainage or intraoperative dural tears.

Most significantly, the current study found a substantial reduction in average pain scores from preoperative to postoperative assessments, indicating meaningful improvement in patients' most critical symptoms. Furthermore, there was a significant increase in lumbar lordosis angle from 34.7 (preoperative) to 42.3 (postoperative). There was also a significant difference between disc height before and after surgery and the effect remained even until the one-year post-operative follow-up as well.

Overall, there is a paucity of studies assessing the clinical or radiological outcomes after performing a unilateral TLIF) procedure; however, it seems that findings from existing studies are in agreement with our study findings despite non-similarity in the outcome measures. For example, the findings of one study showed a reduction in back pain and improved productivity after the procedure [19]. Similarly, evidence suggests that patients undergoing the TLIF stay for a short time in the hospitals, do not have much blood loss, and have less need for blood transfusion compared with combined anterior and posterior fusion [20-22].

The TLIF procedure is given importance over the traditional posterior lumbar interbody fusion (PLIF) because the TLIF provides bilateral anterior column support by one posterolateral approach to the space of the disc [23]. Due to the transforaminal technique, the procedure conserves the anterior and the majority of the posterior longitudinal ligaments, which gives a tension band to compress the graft and averts graft retropulsion [24]. Moreover, the TLIF procedure may be considered an acceptable procedure because the technique does not include excessive soft tissue dissection, thereby preventing scarring and unsteadiness of contiguous ligaments and injury to the adjacent nerve root. These benefits of the TLIF may make the procedure superior to the PLIF, which, unfortunately, has been found to cause neural damage and extensive posterior decompression, resulting in instability [25-27]. These findings are further supported by one review that compared the two procedures, in which the authors found a lower rate of complications, lower mean duration of surgery, and lower blood loss with TLIF than with PLIF [28].

Strengths and limitations

The findings of the current study provide useful insights into the clinical and radiological outcomes of the TLIF procedure. Compared to the similar PLIF technique, TLIF offers some potential advantages such as reduced nerve retraction and better restoration of disc height and lordosis. However, evidence directly comparing TLIF and PLIF outcomes remains limited. These findings contribute to the existing literature on the benefits of TLIF in improving clinical and radiological outcomes and provide a framework for future comparative studies against PLIF. The current study attempted to comprehensively measure outcomes, including estimated blood loss, length of stay, disc height changes, pain reduction, and lumbar lordosis. Pain was assessed using a validated scale. Further research is needed to delineate the advantages of TLIF over other lumbar fusion techniques. 

However, the findings need to be interpreted considering some inherent limitations. First, the study only measured outcomes on a limited sample that was not randomly drawn from all hospitals in Saudi Arabia. This may limit our ability to generalize study findings. Second, our study sample did not have a comparison or control group to compare the TLIF with other procedures, such as PLIF. Hence, the findings cannot claim the superiority of TLIF over other procedures. This may be because our objective was not to present the outcomes of the TLIF to offer it as an acceptable alternative for selected patients. Further, we did not assess patients’ satisfaction with the procedure by assessing their quality of life or productivity; therefore, patients’ perspective is missing in our study. Another limitation of the present study is that long-term clinical outcomes beyond one year were not assessed. Lastly, our study findings may seem favorable because of the healthy sample, as the proportion of diabetes and other comorbidities was lower in our sample. Also, the sample appears to be healthy as there were few smokers in our study, suggesting a favorable study population. 

## Conclusions

The current study findings reveal that the TLIF procedure can be considered a safe procedure to provide anterior and posterior column support by adopting a unilateral posterior approach. The outcomes were favorable in terms of no prolonged length of stay, less blood loss, no mortality, reduction in the severity of pain, and improvement in disc height. However, the appropriate selection of patients for this technique is pivotal for the success of the procedure. Therefore, the patient’s profile and status should be checked before selecting the TLIF procedure. In this study, patients with type 1 MCs on MRI were selected.

Prior studies have found mixed evidence on the relationship between type 1 MCs and postoperative outcomes after lumbar fusion. Further research is needed to delineate if the presence and type of MCs can predict outcomes after TLIF. We recommend collecting more data from different hospitals and assessing the short and long-term outcomes of the TLIF procedure before implementing the procedure for all types of patients. Moreover, a comparison group should be considered in the future to assess the superiority of the procedure over other techniques.
